# Oral administration of *Lacticaseibacillus rhamnosus* HM126 alleviates DNFB-induced atopic dermatitis in BALB/c mice by modulating immunity, gut microbiota, and metabolites

**DOI:** 10.3389/fimmu.2025.1739967

**Published:** 2025-12-29

**Authors:** Lili Xie, Xianping Li, Lu Liu, Junying Zhao, Lingling Luo, Weicang Qiao, Lijun Chen

**Affiliations:** 1 Key Laboratory of Dairy Science, Ministry of Education, Food Science College, Northeast Agricultural University, Harbin, China; 2 National Engineering Research Center of Dairy Health for Maternal and Child, Beijing Sanyuan Foods Co., Ltd., Beijing, China; 3 Beijing Engineering Research Center of Dairy, Beijing Technical Innovation Center of Human Milk Research, Beijing Sanyuan Foods Co., Ltd., Beijing, China

**Keywords:** atopic dermatitis, breast milk-derived probiotics, gut microbiota, *Lacticaseibacillus rhamnosus*, non-targeted metabolomics

## Abstract

**Introduction:**

Probiotics have emerged as a promising and safe alternative therapy for atopic dermatitis (AD) by regulating the gut microbiota-immune axis, correcting type 1/type 2 imbalance, and repairing the skin barrier.

**Methods:**

A mouse model of AD was established using diphenylnitromethane (DNFB). Low, medium, and high doses of human milk-derived *Lacticaseibacillus rhamnosus* HM126 were administered to investigate its effects on the model. We observed the scratching frequency and skin lesion scores after 28 days of continuous oral administration. Serum biochemical indicators and inflammatory cytokines were measured using ELISA, whereas the gut microbiota in feces was analyzed using 16S rDNA sequencing. Non-targeted metabolomics was used to assess the changes in fecal metabolites.

**Results and discussion:**

Compared to the DNFB group, high-dose *L. rhamnosus* HM126 significantly reduced scratching frequency in AD mice. The low-dose group showed significantly reduced IgE levels. Additionally, the IFN-γ/IL-4 ratio significantly increased, indicating that *L. rhamnosus* HM126 modulates type 1/type 2 immune factors toward equilibrium. 16S rDNA analysis revealed that *L. rhamnosus* HM126 significantly reduced the ACE index and Chao 1 index of the gut microbiota in mice with AD, thereby reshaping the composition of the gut microbiome. Metabolomics analysis suggested that *L. rhamnosus* HM126 may improve AD by influencing the levels of asiatic acid, phytosphingosine, Ser-Glu, prostaglandin F2 alpha ethylamide (PGF(2α)EA), argininosuccinic acid, L-rhamnose, and gamma-L-glutamyl-L-glutamic acid. This study demonstrated that *L. rhamnosus* HM126 maintains the type 1/type 2 balance and effectively modifies the gut microbiota structure and metabolic changes to improve AD. Our findings provide a scientific basis for the development of probiotic therapeutics to prevent and treat this condition.

## 1 Introduction

Atopic dermatitis (AD), commonly known as eczema, is a prevalent inflammatory skin disorder. Its hallmark symptoms are recurrent episodes of eczematous skin lesions and refractory pruritus (1, 2). Its prevalence and incidence peak during infancy and early childhood, followed by a second peak in adulthood (3). It profoundly affects patients’ psychosocial functioning and quality of life, and constitutes a major contributor to the global burden of dermatological diseases (1, 4). The pathophysiology of atopic dermatitis is complex, involving multiple contributing factors. Primary causes include genetic predisposition, impaired skin barrier function, and excessive activity of immune cells within the body, which can trigger skin inflammation. Individuals with AD often experience other health issues such as food allergies, asthma, allergic rhinitis (5), and may even face emotional or psychological distress (6). Although type 2 mechanisms are predominate, growing evidence indicates that this disease involves multiple immune pathways.

The homeostasis of the gut microbiota is crucial for human health, and its disruption is a key driver in the onset and progression of various diseases (7), influencing the systemic immune system through bacterial populations and their metabolites. Recent microbiota-related studies have demonstrated a close connection between the gut microbiota and skin homeostasis (8, 9), known as the “gut-skin axis” concept (10). Disruption of the gut microbiota is a key factor driving the onset and progression of autoimmune diseases such as eczema (11). Gut microbiota dysbiosis not only leads to the abnormal secretion of metabolic products and impaired gastrointestinal permeability, but also allows various substances to enter the systemic circulation. These substances trigger immune signaling and neuroendocrine pathways, further inducing epidermal barrier disruption and inflammatory factor accumulation (12). Research indicates that the maternal gut microbiota significantly influences the risk of infant AD through its composition and function (13, 14). Furthermore, indoor microbial communities (such as Cutibacterium and other inflammatory bacteria) and air pollutants (such as PM_10_ and NO_2_) can jointly increase the risk of AD in preschool children through a direct correlation (such as specific microorganisms causing inflammation and pollutants promoting microbial imbalance). Moreover, pollutants can further influence the occurrence of the disease by altering the indoor microbial community (15, 16). Thus, the role of microbiota in AD is multifaceted. Probiotics are live microorganisms that exert beneficial effects on the host health (17, 18). They stimulate type 1 cytokines and suppress the type 2 responses (19). Probiotics enhance epithelial barrier integrity, inhibit pathogen proliferation, restore immune homeostasis, and are emerging as novel therapeutic agents for preventing and treating various allergic diseases, including AD (20).

The DNFB-induced AD mouse model exhibits clinical symptoms highly similar to those observed in AD patients by inducing type 2 immune response-dominated skin inflammation, disrupting skin barrier function, and triggering intense itching. It is widely recognized and applied in preclinical AD research (21, 22). *Lacticaseibacillus rhamnosus* is one of the most extensively studied probiotic strains in pediatric allergy research (23). Previous studies have demonstrated the potential therapeutic role of *Lacticaseibacillus rhamnosus* GG (LGG) in the treatment of AD (24). *L. rhamnosus* HM126 is a beneficial bacterium isolated from human breast milk. This study aims to investigate the effects of *L. rhamnosus* HM126 on immune regulation, gut microbiota composition, and its metabolites in mice exhibiting AD-like symptoms by establishing a mouse model of AD.

## 2 Materials and methods

### 2.1 Screening and cultivation of *L. rhamnosus* HM126

*L. rhamnosus* HM126 was isolated from healthy human breast milk and is currently deposited at the General Microorganism Center of the China General Microorganism Culture Collection Management Committee (CGMCC No. 26167). First, the bacterial strain was cultured at 37 °C for 24 h, followed by two passages to ensure viability. The bacteria were then harvested by centrifugation (6000 rpm, 5 min, 4 °C). After washing to remove residual medium, the pellet was resuspended to the target concentration for subsequent oral administration.

### 2.2 Animals

**Figure 1:**
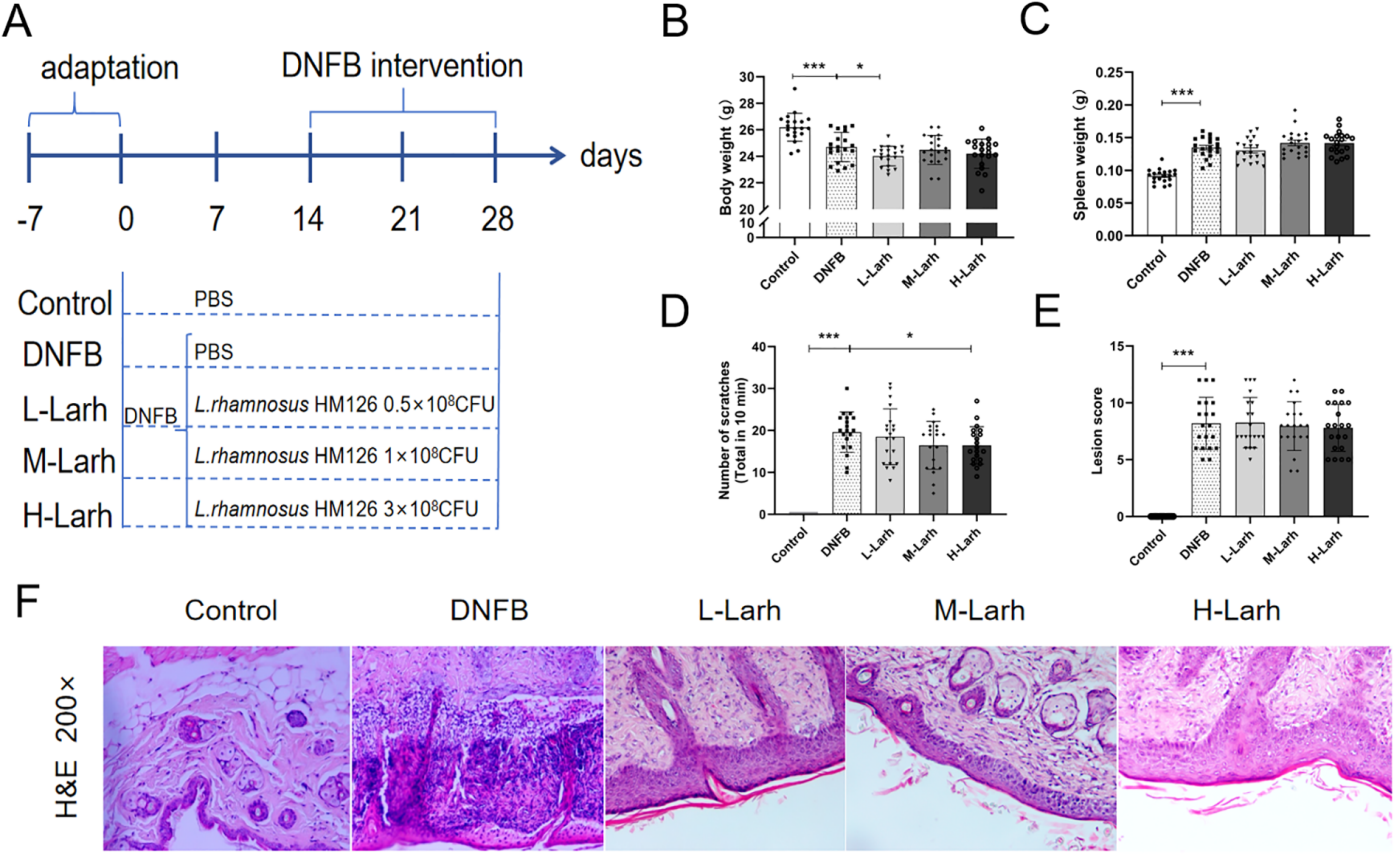
*L. rhamnosus* HM126 alleviates AD-like symptoms in DNFB-treated mice. Mice were administered 0.1 mL PBS or varying doses of *L. rhamnosus* HM126 via oral gavage daily for 28 consecutive days. Except for the control group, mice in all other groups received 100 μL of 0.5% DNFB applied to a shaved area on day 15 for initial sensitization, followed by a booster dose on day 17. On days 21 and 28, 50 μL of 0.5% DNFB solution was applied to the dorsal skin to induce AD. **(A)** Experimental workflow diagram. **(B)** Final body weight. **(C)** Spleen weight. **(D)** Scratching frequency. **(E)** Skin lesion score. **(F)** Representative H&E-stained histopathological changes in mouse skin tissue (H&E 200×). Analysis performed using Dunnett’s multiple comparison test. Data expressed as mean ± SD (n=20). *p<0.05; **p<0.01; ***p<0.001 compared with the DNFB group.

All animal experiments were conducted in accordance with the Beijing Union University Guidelines for the Care and Use of Laboratory Animals and were approved by the Beijing Union University Animal Ethics Committee (JGX11-2306-4; Beijing, China). The experimental workflow is shown in **Figure 1A**. One hundred male BALB/c specific pathogen free (SPF) mice weighing 20–22 g were purchased from Beijing Vital River Laboratory Animal Technology Co., Ltd. (License No.: SCXK(Jing)2021-0006). After 7 days of acclimation feeding, mice were randomly divided into five groups: Control, AD model (DNFB), low-dose (L-Larh, 0.5 × 10^8^ CFU/mouse), medium-dose (M-Larh, 1 × 10^8^ CFU/mouse), and high-dose (H-Larh, 3 × 10^8^ CFU/mouse) (n = 20/group).

### 2.3 Preparation of AD animal models

The Control group received sterile water for 2 weeks, whereas the DNFB group received an equivalent volume of the test sample. Modeling commenced on day 15. One day prior to modeling, hair was removed from a 2 × 2 cm² area on the back of the mouse using 6% sodium sulfide. On day 15, except for the Control group, all groups received 100 μL of 0.5% DNFB (acetone:olive oil = 3:1) applied to the depilated area for sensitization. Re-sensitization occurred on day 17. On days 21 and 28, 50 μL of 0.5% DNFB solution was applied topically to the dorsal skin to induce dermatitis. The Control group received an equal volume of the acetone-olive oil vehicle. Except for the Control and DNFB groups (which received sterile water), all dose groups received 0.1 mL/mouse of the respective test substance via continuous gavage for 28 days.

### 2.4 Skin lesion scoring and scratch number measurement

On day 28, the mice were sensitized with DNFB for 30 min. Following sensitization, the mice were placed individually and observed for 10 min to record the number of times they scratched their trunk and back with their hind paws or bit any part of their body. Consecutive scratching episodes were counted as single occurrences.

On day 29, semi-quantitative scoring of dorsal skin lesions was performed based on the following clinical criteria: erythema, edema/papules, epidermal exfoliation/scratch marks, scaling. Lesions were graded as absent, mild, moderate, or severe (0, 1, 2, or 3 points, respectively), with a maximum total score of 12 points (25).

### 2.5 Spleen measurement and histopathological observation

On day 29, the mice in each group were euthanized via cervical dislocation. The spleen tissue was dissected, and the residual blood was blotted with filter paper and immediately weighed.

The skin samples were excised from the dorsal region. Portions of the skin tissue were fixed in 4% formaldehyde solution, followed by dehydration with graded ethanol, clearing with xylene, paraffin embedding, sectioning at 5 μm thickness, and examination after hematoxylin and eosin (H&E) staining.

### 2.6 ELISA assay for serum IgE, IL-4, IFN-γ, and TGF-β levels

On day 29, blood was collected from the epicardial venous plexus. Serum was obtained via centrifugation at 3000 rpm for 10 min and analyzed. Because cytokine assays require large volumes of serum to ensure accuracy, a random pairing method was employed. Serum from each pair of mice was mixed in equal volumes for cytokine detection and each group of 20 mice yielded 10 pooled serum samples (n=10/group).

### 2.7 16S rDNA sequencing and data analysis

Fresh fecal samples were collected and stored at -80 °C (Control, DNFB, and H-Larh groups; n=10/group). Sequencing and data analysis were performed by Shanghai Applied Protein Technology Co., Ltd. Genomic DNA was extracted using the Omega Soil DNA Kit. The V3-V4 region of the 16S rDNA gene was amplified via PCR and library preparation, followed by sequencing on the Illumina platform. Data were processed using QIIME 2 and DADA2 workflows to obtain ASV and species annotations (based on the Silva database). Subsequently, α and β diversity analyses were conducted, and LEfSe was employed to identify differentially abundant species between groups (26).

### 2.8 Metabolomics analysis of fecal samples

Collected feces were rapidly frozen in liquid nitrogen and stored at -80 °C (Control, DNFB, and H-Larh groups; n=10/group). Non-targeted metabolomics analysis was performed by Shanghai Applied Protein Technology Co., Ltd. (Shanghai, China) In brief, samples were extracted with cold methanol/acetonitrile/water solution (2:2:1, v/v), subjected to low-temperature ultrasonication and centrifugation, followed by vacuum drying of the supernatant. After redissolution and centrifugation, the supernatant was transferred to injection vials for analysis. After separation by a Vanquish LC ultra-high-performance liquid chromatography (UHPLC) system, mass spectrometry analysis was performed using a Q Exactive series mass spectrometer (Thermo Fisher Scientific, Waltham, MA, USA). Column temperature was set at 25 °C, with acetonitrile-water gradient elution. Mass spectrometry scanned in both positive and negative ion modes, covering a range of 80–1200 Da. Raw data were processed using ProteoWizard and XCMS software to complete peak extraction and alignment. Subsequent steps included metabolite identification, data preprocessing, and quality assessment. Orthogonal Partial Least Squares Discriminant Analysis (OPLS-DA) was employed to analyze metabolic changes. Differentially expressed metabolites were screened using VIP > 1 and *p* < 0.05, followed by pathway enrichment analysis via MetaboAnalyst (https://www.metaboanalyst.ca/).

### 2.9 Statistical analysis

Statistical analysis was performed using GraphPad Prism 10.1.2 software (San Diego, CA, USA). One-way analysis of variance was conducted, followed by Dunnett’s multiple comparison test to analyze the differences in body weight, spleen weight, scratching frequency, skin lesion score, inflammatory factors, and mast cells. The data are presented as mean ± standard deviation (SD). Spearman correlation analysis between gut microbiota and differential metabolites. The significance level was set at *p* < 0.05.

## 3 Results

### 3.1 Protective effect of *L. rhamnosus* HM126 on skin lesions in AD mice

To investigate the effects of the model establishment and HM126 intervention on body weight, we selected male mice with balanced body weights prior to experimentation. Post-experiment, significant differences in body weight were observed between the DNFB group and the Control group (*p* < 0.001) (**Figure 1B**), indicating that DNFB-induced dermatitis affects mouse body weight. Compared to the DNFB group, the L-Larh group exhibited significantly reduced body weight (*p* = 0.027).

The spleen is the most important immune organ in the body. Compared with the control group, the spleen weight significantly increased in the DNFB group after the experiment (*p* < 0.001). Compared to the DNFB group, no significant differences in spleen weight were observed among all dose groups after the experiment (*p* > 0.05) (**Figure 1C**). Reduced scratching behavior indicates the alleviation of allergic pruritus. Compared to the DNFB group, all dose groups showed a trend toward reduced scratching frequency during modeling, with the H-Larh group exhibiting the lowest frequency and significant differences (*p = 0.039*) (**Figure 1D**). Compared to the DNFB group, none of the dose groups showed significant differences in skin lesion scores after treatment (*p* > 0.05) (**Figure 1E**).

Dorsal skin tissue was collected for H&E staining to evaluate symptoms in mice with DNFB-induced AD. As shown in **Figure 1F**, the dorsal skin from the Control group exhibited intact structures in the epidermis, appendages, dermis, and subcutaneous tissue, with no evidence of damaging alterations or inflammatory cell infiltration. In contrast, the DNFB group exhibited typical allergic reactions, including epidermal erosion of the dorsal skin that progressed to ulceration, partial or complete loss of the epidermal cell layer, and basement membrane disruption. Extensive infiltration of inflammatory cells (including polymorphonuclear leukocytes) resulted in the formation of pustules and crusts. Individual specimens exhibited epidermal necrosis (full-thickness type) separated from the dermis, revealing a strongly eosinophilic cytoplasm within the necrotic foci. Macrophage dermal infiltration and epidermal hyperplasia are the hallmark features of animal models of AD. This demonstrates the validity of the AD mouse model employed in this experiment.

### 3.2 *L. rhamnosus* HM126 treatment reduces inflammatory responses in AD mice

The imbalance in type 1/type 2 cell subsets, which leads to a series of cytokine alterations, constitutes the primary immunological mechanism in AD pathogenesis (27, 28). Type 1 cells, characterized by IFN-γ secretion, drive cellular immunity, cytotoxic responses, and delayed-type hypersensitivity. In contrast, type 2 cells, defined by IL-4 production, promote humoral immunity and contribute to (atopic/allergic) hypersensitivity. In AD, type 2-driven IL-4 expression activates B cells to produce excess IgE antibodies. External antigens and allergens absorbed through the skin bind to IgE molecules on mast cell surfaces, triggering mast cell degranulation and release of histamine and other mediators. This process induces pruritus and perpetuates type 2 cell dysregulation. Furthermore, Evidence suggests that TGF-β ameliorates AD by suppressing TNF-α and IgE secretion.

**Figure 2:**
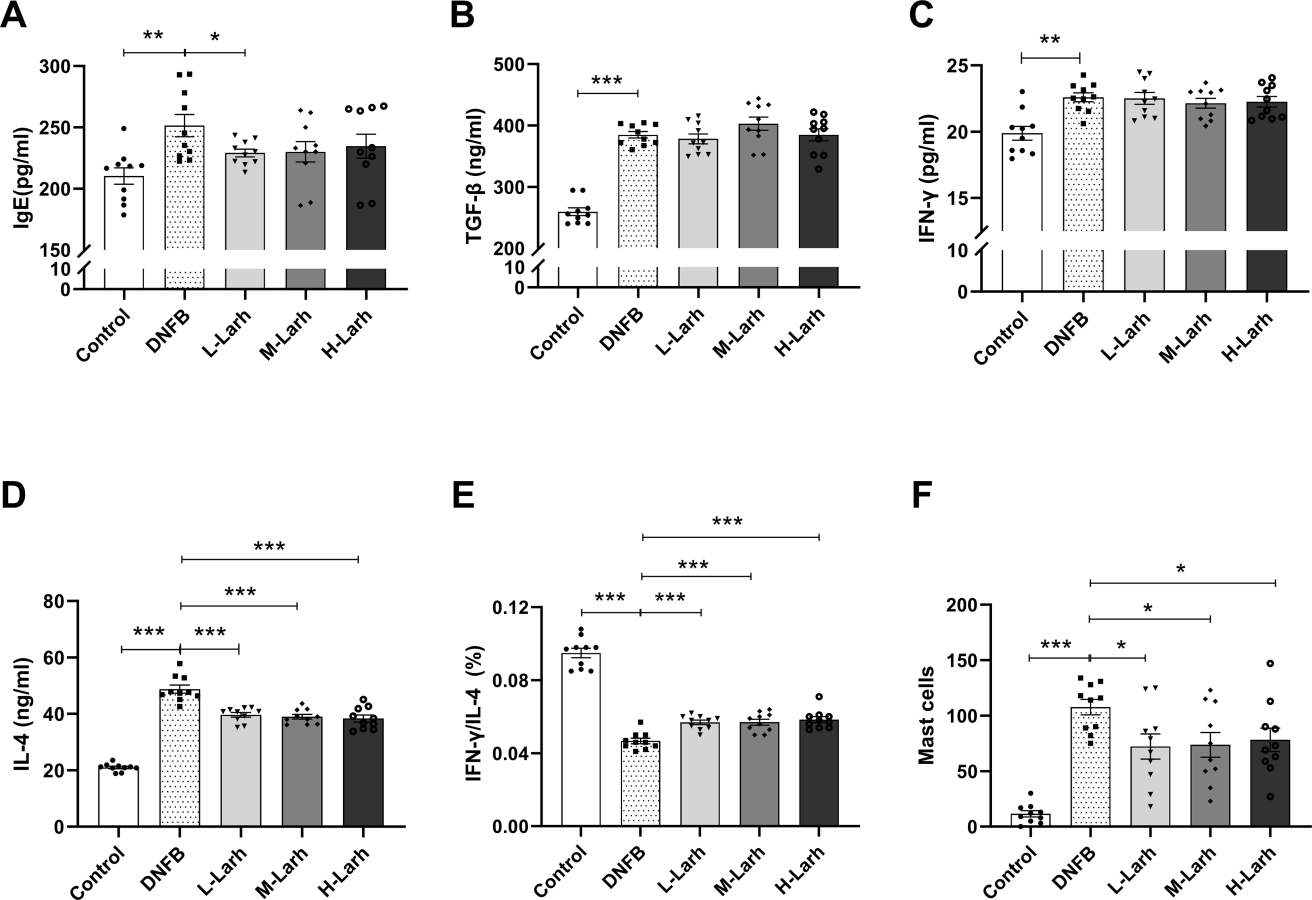
*L. rhamnosus* HM126 reduces inflammatory responses in AD mice. **(A–D)** Serum cytokines IgE, TGF-β, IFN-γ, IL-4. **(E)** Serum IFN-γ/IL-4 ratio. **(F)** Mast cells. Analysis was performed using Dunnett’s multiple comparison test. Data were expressed as the mean ± SD (n=10). *p < 0.05; **p < 0.01; ***p < 0.001 compared to the DNFB group.

**Figures 2A–E** illustrates the effects of *L. rhamnosus* HM126 treatment on cytokine expression in the serum of AD mice. Compared with the Control group, serum cytokine levels in DNFB group exhibited altered patterns, with extremely significant increases in TGF-β (*p* < 0.001), IFN-γ (*p* = 0.003), IgE (*p* = 0.002), and IL-4 (*p* < 0.001). Compared with the DNFB group, the IgE levels in the L-Larh group decreased significantly (*p* = 0.032).All three HM126 dose groups exhibited potent, non-dose-dependent downregulation of the type 2-associated cytokine IL-4 (*p* < 0.001). *L. rhamnosus* HM126 did not affect type 1-related cytokine IFN-γ levels (*p* > 0.05), and TGF-β levels in all dose groups showed no significant difference compared to those in the DNFB mice (*p* > 0.05). The IFN-γ/IL-4 ratio in serum significantly increased across all dose groups (*p* < 0.001), suggesting that *L. rhamnosus* HM126 intervention improved the type 1/type 2 imbalance in AD mice. Compared to the Control group, the DNFB group showed significantly higher number of skin mast cells (*p* < 0.001). Compared to DNFB group, the number of skin mast cells in all dose groups was significantly reduced (*p* = 0.016, *p* = 0.019, *p* = 0.032) (**Figure 2F**).

### 3.3 Effects of *L. rhamnosus* HM126 on the gut microbiome of AD mice

**Figure 3:**
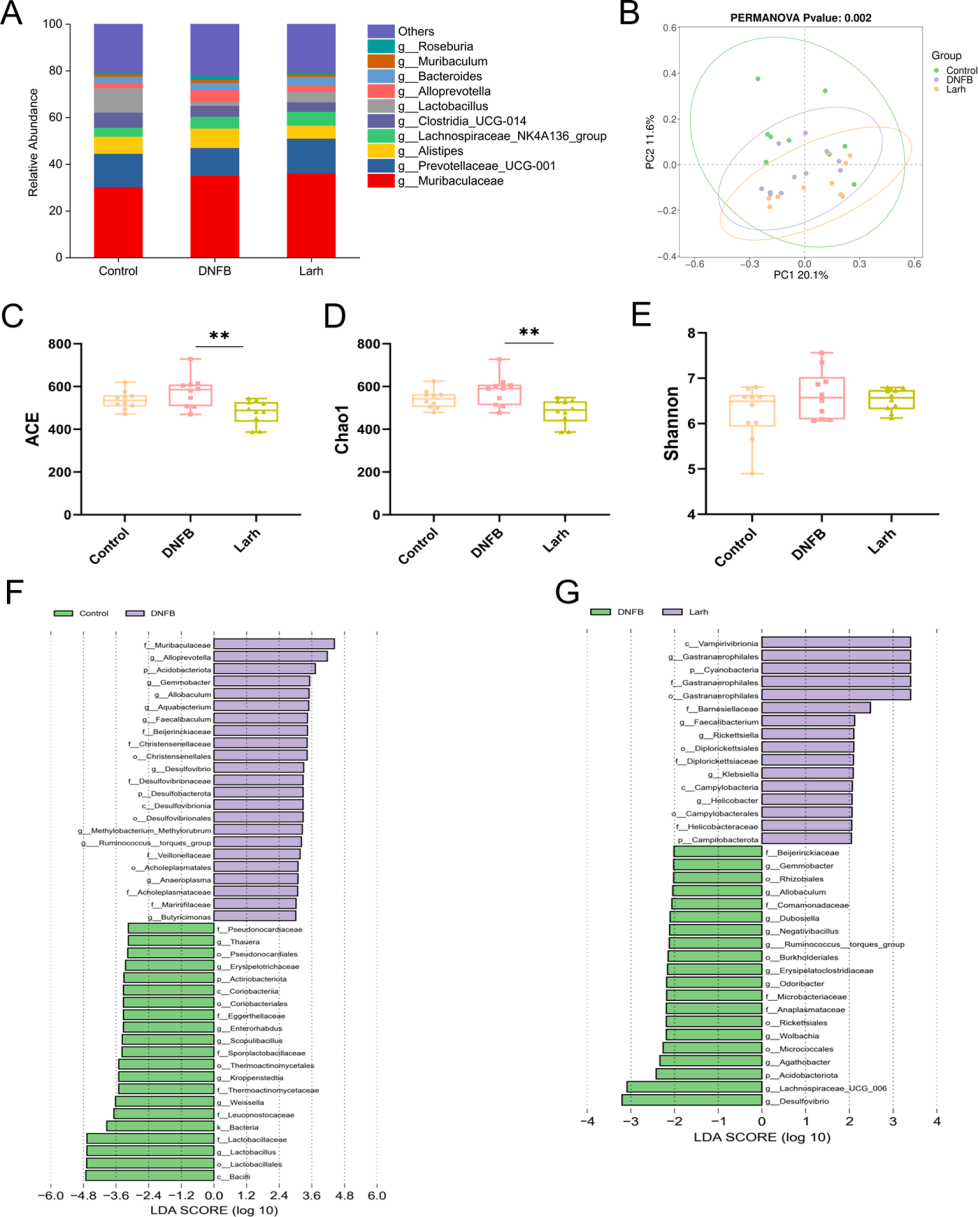
Effects of *L. rhamnosus* HM126 on the gut microbiota of AD mice. **(A)** Comparison of relative genus abundance. **(B)** β-diversity based on Bray-Curtis distance. **(C–E)** Boxplots of intergroup differences in α-diversity indices. **(F, G)** Analysis of intergroup differences in species.

To evaluate the impact of *L. rhamnosus* HM126 intervention on gut microbiota diversity and abundance in DNFB mice, we performed 16S rDNA gene sequencing of colonic contents. Sequencing depth assessment results indicate that the rarefaction curves for all samples have flattened (**Supplementary Figure S1**), demonstrating that the current sequencing depth has captured the vast majority of microbial species present in the samples. Genus-level analysis revealed a similar composition of dominant bacterial groups across all samples, including *Muribaculaceae*, *Prevotellaceae* UCG-001, *Alistipes*, *Lachnospiraceae* NK4A136 group, *Clostridia* UCG-014, *Lactobacillus*, *Alloprevotella*, *Bacteroides*, *Muribaculum*, and *Roseburia*. Compared to the DNFB group, *L. rhamnosus* HM126 intervention increased the abundance of *Prevotellaceae*_UCG-001 and *Lactobacillus* and decreased the abundance of *Alistipes*, *Alloprevotella*, *Muribaculum*, and *Roseburia* (**Figure 3A**). Collectively, these findings provide evidence that *L*. *rhamnosus* HM126 exerts a regulatory effect on the intestinal microbiota of mice.

Changes in α-diversity within gut microbiota were characterized using the Shannon index, ACE index, and Chao1 index (**Figures 3C–E**). Notably, the ACE (*p* = 0.005) and Chao1 (*p* = 0.004) indices were substantially lower in the *L. rhamnosus* HM126 group than in the DNFB group. No notable differences in the Shannon index were observed among groups. A significant disparity in microbial community structure among the Control, DNFB, and Larh groups was observed, as determined by Bray-Curtis beta-diversity (PERMANOVA, *p* = 0.002), (**Figure 3B**),indicating that *L. rhamnosus* HM126 intervention substantially influenced gut microbiota composition. In terms of distribution trends, the DNFB group showed a more pronounced separation from the control group, suggesting that the AD model may exacerbate disease progression by altering gut microbiota homeostasis. The sample distribution of the Larh group fell between the two extremes, partially aligning with the control group, implying that probiotics may partially reverse AD-associated dysbiosis by modulating the microbial structure.

LEfSe analysis revealed that the DNFB group showed a marked enrichment of the harmful bacterium Desulfovibrio. (**Figures 3F, G**). *Desulfovibrio*, Desulfovibrionaceae, and Desulfovibrionales, all related to the Desulfovibrionaceae family, can produce hydrogen sulfide through sulfate-reducing metabolism, disrupt intestinal barrier integrity, and lead to the activation of pro-inflammatory signaling pathways such as NF-κB, which correlates with the type 2 immune shift in AD. In contrast, *L. rhamnosus* HM126 intervention significantly enriched beneficial bacteria such as *Faecalibacterium* and *Lactobacillus* and partially reversed dysbiosis. In summary, *L. rhamnosus* HM126 may offer a novel therapeutic strategy for AD by reshaping the gut microbiota, suppressing opportunistic pathogens, and enriching beneficial metabolic bacteria.

### 3.4 Effects of *L. rhamnosus* HM126 on fecal metabolites in AD mice

**Figure 4:**
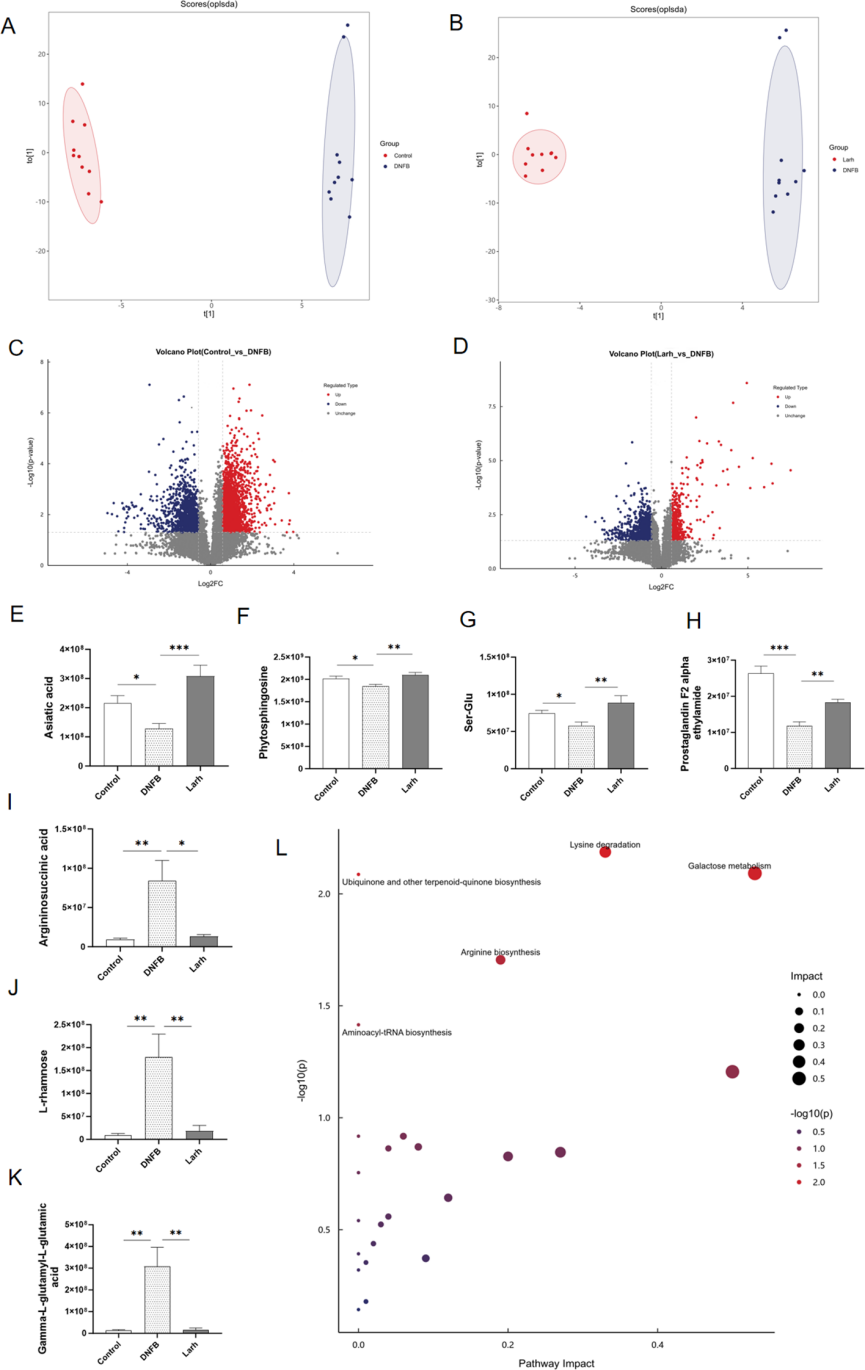
Effects of *L. rhamnosus* HM126 on fecal metabolites in AD mice. **(A–B)** OPLS-DA results of mouse fecal metabolites. **(C, D)** Volcano plots. **(E–K)** Effects of *L. rhamnosus* HM126 intervention on fecal metabolites in atopic dermatitis mice. Analyzed by unpaired t-tests, *p < 0.05, **p < 0.01, ***p < 0.001. **(L)** Metabolic pathways associated with fecal samples from AD mice after *L. rhamnosus* HM126 intervention.

To further investigate the effects of *L. rhamnosus* HM126 on fecal metabolites in mice with AD, non-targeted metabolomic analysis was performed on fecal samples from the Control, DNFB, and H-Larh groups. PCA analysis indicates that QC samples cluster closely together, demonstrating good reproducibility of the experiment (**Supplementary Figure S2**). We constructed an OPLS-DA model for fecal samples to characterize intergroup metabolic changes (**Figures 4A, B**). Differentially expressed metabolites were screened using the criteria of VIP> 1 and *p* < 0.05. Compared with the control group, the DNFB group showed 325 differential metabolites, including 122 upregulated and 203 downregulated ones. Additionally, 134 differential metabolites were identified between the DNFB group and the Larh group, with 53 upregulated and 81 downregulated in the Larh group relative to the DNFB group. Integrative analysis of metabolomic data from the model and probiotic intervention groups revealed that these differential metabolites were mainly categorized into lipids, organic acids, organic heterocyclic compounds, and aromatic compounds. Notably, the number of downregulated metabolites was consistently higher than that of upregulated ones across the comparative groups (**Figures 4C, D**).

Further screening of metabolites present in both the DNFB vs Control and Larh vs DNFB comparisons identified 33 differential metabolites that met these criteria, potentially representing the core targets of *L. rhamnosus* HM126 intervention (**Supplementary Table S1**). Following *L. rhamnosus* HM126 treatment, significant metabolic rebalancing was observed for certain metabolites. Specifically, asiatic acid, phytosphingosine, Ser-Glu, PGF(2α)EA levels were significantly reduced and significantly increased in the DNFB and Larh groups, respectively. Argininosuccinic acid, L-rhamnose, and gamma-L-glutamyl-L-glutamic acid were significantly upregulated in the model group and significantly downregulated after *L. rhamnosus* HM126 treatment. (**Figures 4E–K**) These substances may be associated with the improvement of *L. rhamnosus* HM126 in mice.

Based on the differentially identified metabolites in fecal samples, metabolic pathway analysis using MetPA revealed 25 pathways potentially related to AD metabolic dysregulation. According to the significance criteria *p* < 0.05 and impact > 0.1, the following pathways were most likely associated with the mechanism by which *L. rhamnosus* HM126 alleviated AD. Three pathways—lysine degradation, galactose metabolism, and arginine biosynthesis—were disrupted by *L. rhamnosus* HM126 to improve endogenous metabolism in AD (**Figure 4L**).

### 3.5 Correlation analysis among gut microbiota and metabolites

**Figure 5:**
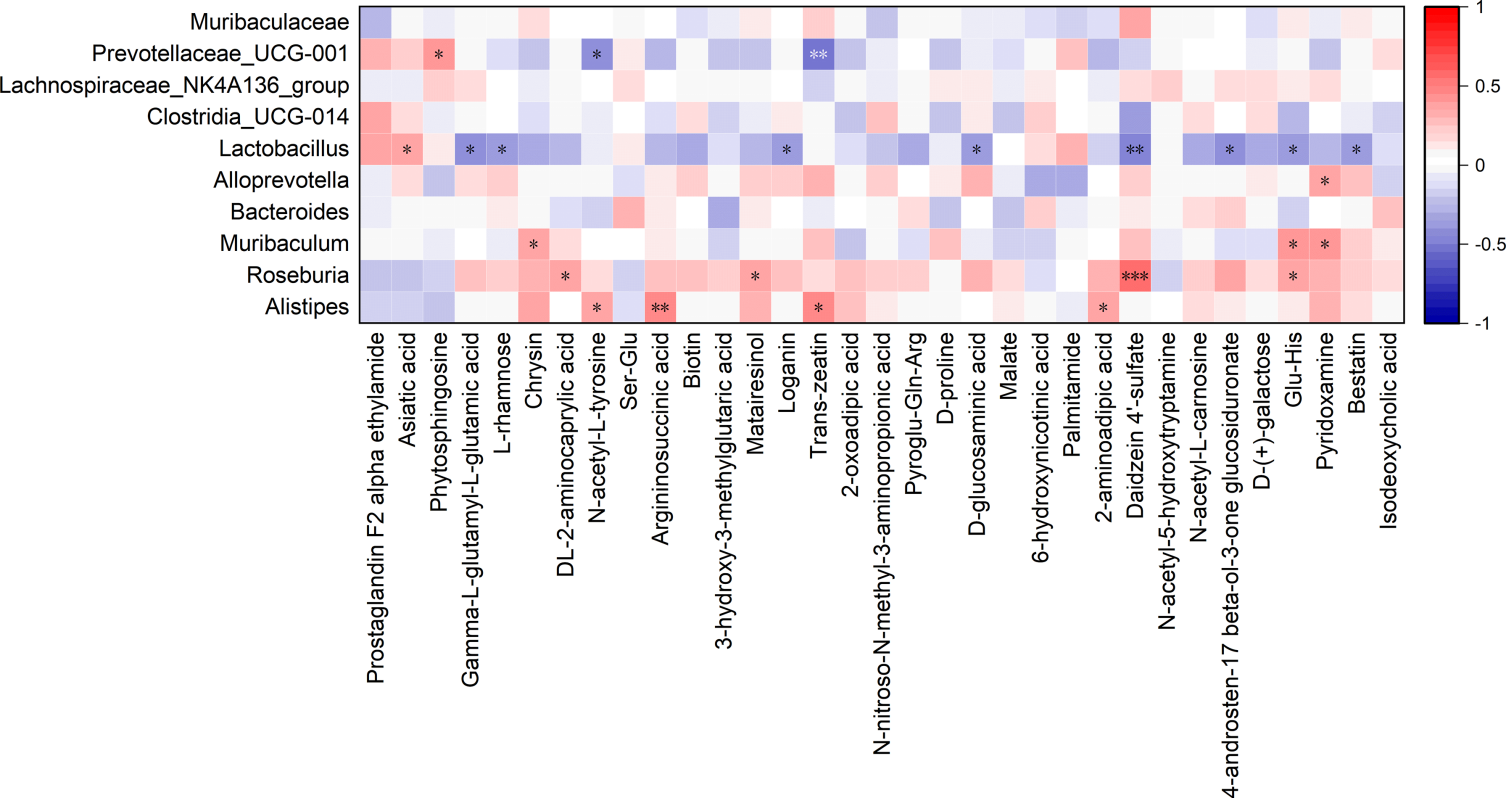
Spearman correlation analysis between gut microbiota and differential metabolites. * *p* < 0.05, ** *p* < 0.01, *** *p* < 0.001.

To further elucidate the associations between gut microbiota and metabolites, Spearman correlation analyses were conducted between differentially expressed metabolites and gut microbiota (**Figure 5**). The key gut bacterial genus *Prevotellaceae* UCG-001 positively correlated with phytosphingosine and negatively correlated with N-acetyl-L-tyrosine and trans-zeatin. *Alistipes* was positively correlated with N-acetyl-L-tyrosine, argininosuccinic acid, trans-zeatin, and 2-aminoadipic acid. A positive correlation of *Lactobacillius* with asiatic acid and negative correlations with L-rhamnose, D-glucosaminic acid, Glu-His, and other compounds were observed. Additionally, *Roseburia* was positively correlated with DL-2-aminocaprylic acid, Materia resinol, Daidzein 4’-sulfate, and Glu-His.

## 4 Discussion

This study indicates that intervention with *L. rhamnosus* HM126 can alleviate AD symptoms by modulating immune responses and gut microbiota. *L. rhamnosus* HM126 intervention markedly reduced serum IL-4 and IgE expression levels in AD mice, marking a shift away from type 2-polarized immunity and contributing to the restoration of the immune balance between the type 1 and type 2 pathways. Furthermore, HM126 treatment significantly reduced mast cell numbers in the skin tissues of DNFB-induced AD model mice, suggesting that it may alleviate skin inflammation by regulating mast cell-mediated inflammatory responses. Our study found that after intervention with *L. rhamnosus* HM126, while the scratching frequency of mice was significantly reduced, no obvious change was observed in the skin lesion score during the same period. Given that the structural repair process of skin tissue is relatively slow, a prolonged intervention period may be required to achieve visible improvements. This result is consistent with the study by Kim et al. (29).

Intake of *L. rhamnosus* HM126 reshaped the body’s microbial ecology. Probiotics leverage the regulatory power of gut microbiota to mitigate allergic susceptibility (30). The present experiment revealed that the ACE and Chao1 indices were significantly lower in the *L. rhamnosus* HM126 intervention group compared to the DNFB group, with markedly different β-diversity in the gut microbiota. This may be attributed to *L. rhamnosus* HM126, which successfully colonizes the gut (31), where it regulates the microbial community composition through competitive niche occupation. This action suppresses certain overproliferating or conditionally pathogenic bacterial groups, thereby reducing the overall species richness within the community. Following *L. rhamnosus* HM126 intervention, we observed elevated proportions of *Lactobacillus* and *Prevotellaceae*_UCG-001 in the mouse gut communities, along with decreased levels of *Alistipes* and *Alloprevotella. Prevotellaceae*_UCG-001, a gram-negative anaerobe, is the primary butyrate-producing bacterium in the gut microbiota (32). Butyrate is a crucial short-chain fatty acid (SCFA) that regulates the gut microbiota balance, maintains intestinal barrier integrity, and modulates immune responses (33, 34). Biagi et al. noted that *Lactobacillus* is a key probiotic in the animal gut microenvironment, participating in microbial balance regulation, and enhancing immunity and disease resistance (35). In summary, *L. rhamnosus* HM126 exerts regulatory effects on the intestinal microbiota of mice, although its specific mechanisms warrant further investigation.

Fecal metabolites are considered co-metabolites of the gut microbiota and host, reflecting not only the state of the gut microbiota but also serving as a bridge between gut microbes and the host (36). Metabolomics, a vital component of systems biology, provides a more accurate reflection of biological changes (37, 38). Following HM126 intervention in AD mice, differential metabolites, including asiatic acid, phytosphingosine, Ser-Glu, and PGF(2α)EA were upregulated in fecal metabolites. In contrast, the levels of argininosuccinic acid, L-rhamnose, and gamma-L-glutamyl-L-glutamic acid were downregulated. Asiatic acid exhibits anti-inflammatory activity, and Moon et al. (39) demonstrated that asiatic acid treatment inhibited the production of type 1-, type 2-, and inflammatory cytokines, and hindered the NF-κB and MAPK signaling pathways underlying their expression. Additionally, asiatic acid suppressed DNCB-induced AD skin lesions and other AD features in mice (39). Ceramide levels are reduced in AD patients (40). Phytosphingosine serves not only as a key building block for a healthy skin barrier, but also as an indispensable precursor in ceramide synthesis. Phytosphingosine promotes collagen synthesis, enhances skin cell adhesion and tightness, and facilitates skin barrier repair (41). It exerts anti-inflammatory effects by inhibiting protein kinase C (PKC) enzyme activity (42), thereby alleviating symptoms such as skin redness and itching. Phytosphingosine has been demonstrated to reduce the release of proinflammatory factors (IL-6, IL-17A) and modulate the immune response by inhibiting NF-κB, JAK/STAT, and MAPK signaling pathways (43). Arginine metabolism is a key metabolic factor regulating macrophage polarization and inflammatory responses. Argininosuccinic acid, an intermediate product of the urea cycle, can supply arginine. Arginine is then converted by arginase 1 (ARG1) to yield ornithine and polyamines. The latter participate in cell proliferation and tissue repair, and are closely associated with M2-type macrophage function (44).

This study observed that *L. rhamnosus* HM126 significantly reduced scratching episodes in AD mice, downregulated serum IL-4 and IgE levels, modulated type 1/type 2 balance, and improved AD. Multiple clinical studies have confirmed that *Lacticaseibacillus rhamnosus* effectively improves the severity score (SCORAD) of pediatric AD and modulates host immunity (45). This study provides evidence for *L. rhamnosus* HM126 as a potential probiotic supplement for human AD treatment. Currently, the efficacy of probiotics in human AD therapy remains controversial. This debate likely stems from differences in the immunomodulatory mechanisms of various probiotics, subject heterogeneity, and variations in intervention protocols, leading to inconsistent clinical outcomes. For instance, research by Cukrowska B et al. (46) demonstrated that specific probiotic combinations are effective for children with AD accompanied by cow’s milk protein allergy (CMPA). This also suggests that AD combined with food allergies (such as CMPA) may represent a target population for probiotic intervention. Probiotics also hold potential application value in other allergic diseases. Li et al. (47) found that *Lactobacillus reuteri* CCFM1072 and CCFM1040 significantly reduced serum IgE levels in allergic asthma model mice while modulating gut microbiota composition, thereby effectively alleviating airway inflammation. Similarly, another study (48) reported that *Bifidobacterium longum subspecies infantis* CCFM111 not only significantly suppressed IgE and type 2 cytokine expression (including IL-4, IL-13, and IL-6) in allergic rhinitis models but also increased the abundance of beneficial bacteria such as Akkermansia and Ruminiclostridium. This further confirms the potential of probiotics in systemic immune regulation and gut microbiome restoration.

This study has several limitations. First, species differences and the complexity of AD pathogenesis (e.g., genetic and environmental factors) mean that animal models cannot fully replicate human disease. Second, the 4-week short-term intervention only revealed rapid modulation of IL-4, IgE, and scratching behavior by *L. rhamnosus* HM126, failing to assess its long-term reparative effects on skin structure. Furthermore, 16S rDNA sequencing resolution is limited to the genus level, making precise species identification challenging. The specific key species mediating the alleviation of AD require further clarification.

## 5 Conclusion

This study employed a mouse model of AD, 16S rDNA gut microbiota sequencing, and non-targeted metabolomics to investigate the effects of *L. rhamnosus* HM126 on DNFB-induced AD mice. Results indicate that this probiotic effectively alleviates symptoms in AD mice, reduces serum IgE, IL-4 levels and mast cell counts, and modulates the IFN-γ/IL-4 ratio. 16S rDNA sequencing and metabolomic studies revealed that HM126 improves gut microbiota dysbiosis in AD mice, potentially exerting its anti-atopic effects by modulating metabolic pathways through alterations in metabolic compounds. These findings substantiate the mechanism by which probiotics improve AD and provide a scientific reference for the development of probiotic therapeutics to prevent and treat this condition. Based on these findings, future research should focus on validating its efficacy in human populations and elucidating its precise molecular mechanisms.

## Data Availability

The data presented in the study are deposited in the National Center for Biotechnology Information (NCBI) repository, accession number PRJNA1375494.
