# Supplementary material for: Oral administration of Lacticaseibacillus rhamnosus HM126 alleviates DNFB-induced atopic dermatitis in BALB/c mice by modulating immunity, gut microbiota, and metabolites
Source: Front Immunol. 2025 Dec 29;16:1739967. doi: 10.3389/fimmu.2025.1739967 (PMC12791040; doi:10.3389/fimmu.2025.1739967)
Supplement: Supplementary file 1 [file Table1.docx]

Supplementary Material

# Supplementary Figures and Tables

For more information on Supplementary Material and for details on the different file types accepted, please see [here](https://www.frontiersin.org/guidelines/author-guidelines#supplementary-material).

## Supplementary Figures

##
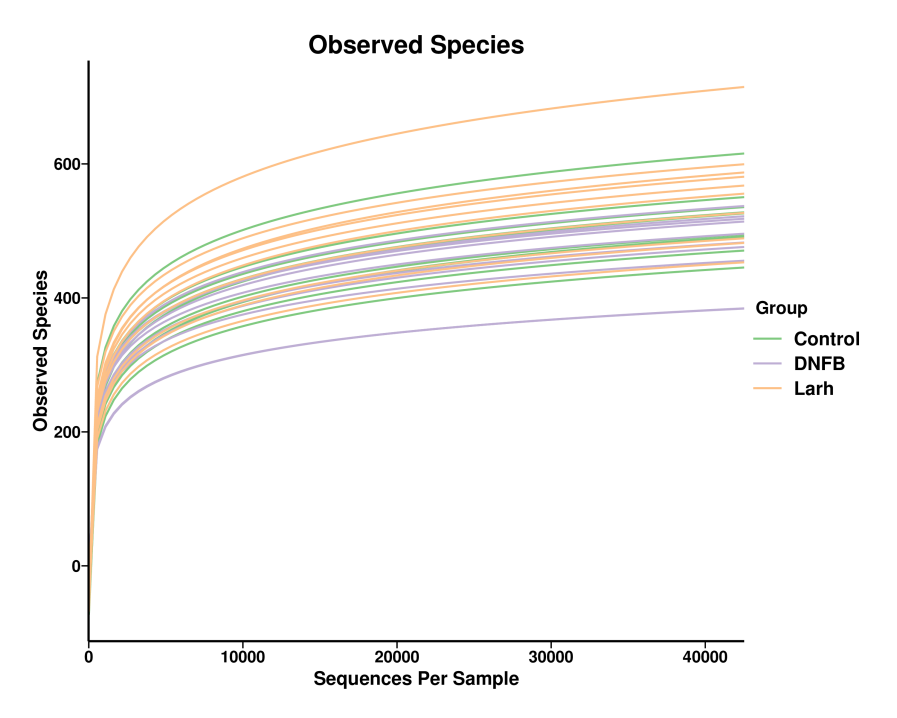


## Supplementary Figure 1.Species-specific 16S rDNA sequencing rarefaction curves for each sample.


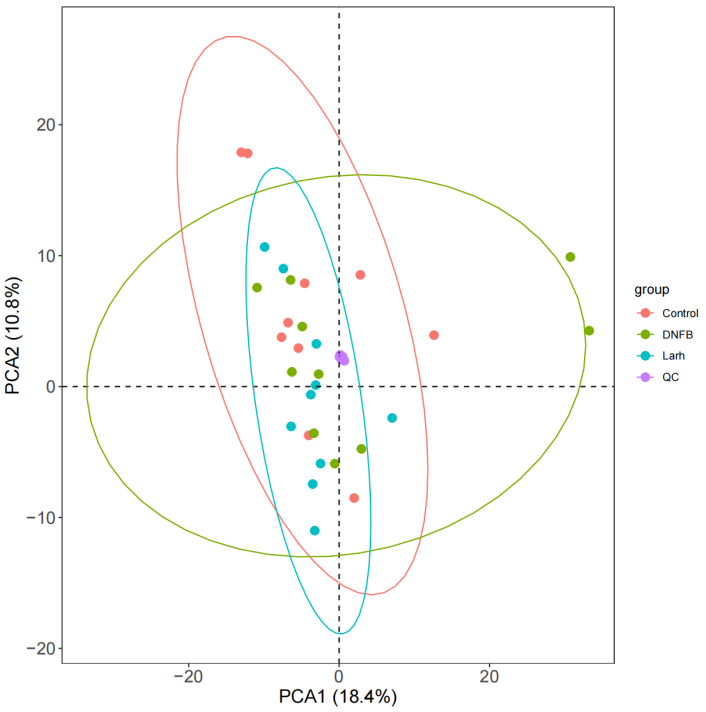


## Supplementary Figure 2.Species-specific 16S rDNA sequencing rarefaction curves for each sample.

## Supplementary Tables

**Supplementary Table S1.** Effects of HM126 on differential metabolites in mice with atopic dermatitis.

| No. | Name | m/z | rt(s) | Foldchange | *p*-value | VIP | ESI | Trend | |
| --- | --- | --- | --- | --- | --- | --- | --- | --- | --- |
|  |  |  |  |  |  |  |  | DNFBvsControl | LarhvsDNFB |
| 1 | 6-hydroxynicotinic acid | 140.03 | 398.95 | 1.17 | 0.0337 | 1.07 | + | ↓ | ↑ |
| 2 | Asiatic acid | 523.30 | 38.75 | 2.40 | 0.0004 | 5.08 | - | ↓ | ↑ |
| 3 | Isodeoxycholic acid | 415.28 | 180.64 | 1.66 | 0.0108 | 2.36 | + | ↓ | ↑ |
| 4 | N-acetyl-5-hydroxytryptamine | 241.12 | 368.10 | 1.39 | 0.0432 | 1.26 | + | ↓ | ↑ |
| 5 | Palmitamide | 256.26 | 43.22 | 1.27 | 0.0350 | 1.40 | + | ↓ | ↑ |
| 6 | Phytosphingosine | 318.30 | 67.67 | 1.14 | 0.0020 | 7.48 | + | ↓ | ↑ |
| 7 | Prostaglandin F2 alpha ethylamide | 364.30 | 276.29 | 1.56 | 0.0002 | 1.05 | + | ↓ | ↑ |
| 8 | Ser-Glu | 235.11 | 370.07 | 1.53 | 0.0095 | 1.98 | + | ↓ | ↑ |
| 9 | 2-aminoadipic acid | 144.08 | 253.26 | 0.14 | 0.0378 | 1.62 | + | ↑ | ↓ |
| 10 | 2-oxoadipic acid | 159.03 | 231.50 | 0.75 | 0.0247 | 1.01 | - | ↑ | ↓ |
| 11 | 3-hydroxy-3-methylglutaric acid | 161.05 | 378.98 | 0.64 | 0.0206 | 1.51 | - | ↑ | ↓ |
| 12 | 4-androsten-17 beta -ol-3-one glucosiduronate | 487.23 | 347.59 | 0.11 | 0.0454 | 1.52 | + | ↑ | ↓ |
| 13 | Argininosuccinic acid | 291.12 | 290.11 | 0.16 | 0.0126 | 2.68 | + | ↑ | ↓ |
| 14 | Bestatin | 331.17 | 451.81 | 0.16 | 0.0488 | 1.26 | + | ↑ | ↓ |
| 15 | Biotin | 227.09 | 282.26 | 0.14 | 0.0162 | 13.12 | + | ↑ | ↓ |
| 16 | Chrysin | 255.07 | 166.29 | 0.44 | 0.0070 | 1.37 | + | ↑ | ↓ |
| 17 | D-(+)-galactose | 215.04 | 58.93 | 0.24 | 0.0459 | 3.60 | - | ↑ | ↓ |
| 18 | D-glucosaminic acid | 196.08 | 252.04 | 0.16 | 0.0315 | 5.58 | + | ↑ | ↓ |
| 19 | D-proline | 114.06 | 337.20 | 0.75 | 0.0303 | 3.46 | - | ↑ | ↓ |
| 20 | Daidzein4'-sulfate | 333.01 | 24.75 | 0.61 | 0.0426 | 4.73 | - | ↑ | ↓ |
| 21 | DL-2-aminocaprylic acid | 160.13 | 253.22 | 0.42 | 0.0087 | 6.40 | + | ↑ | ↓ |
| 22 | Gamma-L-glutamyl-L-glutamic acid | 259.10 | 283.25 | 0.05 | 0.0039 | 5.58 | + | ↑ | ↓ |
| 23 | Glu-His | 285.08 | 48.04 | 0.54 | 0.0479 | 3.78 | + | ↑ | ↓ |
| 24 | L-rhamnose | 187.07 | 212.18 | 0.10 | 0.0062 | 4.04 | + | ↑ | ↓ |
| 25 | Loganin | 413.16 | 325.93 | 0.19 | 0.0209 | 1.74 | + | ↑ | ↓ |
| 26 | Malate | 133.01 | 432.51 | 0.61 | 0.0336 | 1.19 | - | ↑ | ↓ |
| 27 | Matairesinol | 341.13 | 290.76 | 0.11 | 0.0206 | 2.86 | + | ↑ | ↓ |
| 28 | N-acetyl-L-carnosine | 269.12 | 348.14 | 0.20 | 0.0445 | 2.98 | + | ↑ | ↓ |
| 29 | N-acetyl-L-tyrosine | 180.07 | 325.53 | 0.57 | 0.0088 | 3.83 | - | ↑ | ↓ |
| 30 | N-nitroso-N-methyl-3-aminopropionic acid | 133.07 | 212.25 | 0.14 | 0.0255 | 2.54 | + | ↑ | ↓ |
| 31 | Pyridoxamine | 151.10 | 241.41 | 0.25 | 0.0486 | 2.55 | + | ↑ | ↓ |
| 32 | Pyroglu-Gln-Arg | 414.20 | 203.42 | 0.29 | 0.0255 | 1.85 | + | ↑ | ↓ |
| 33 | Trans-zeatin | 220.11 | 239.50 | 0.46 | 0.0243 | 1.51 | + | ↑ | ↓ |
